# The Impact of Obstructive Sleep Apnea on Metabolic and Inflammatory Markers in Consecutive Patients with Metabolic Syndrome

**DOI:** 10.1371/journal.pone.0012065

**Published:** 2010-08-11

**Authors:** Luciano F. Drager, Heno F. Lopes, Cristiane Maki-Nunes, Ivani C. Trombetta, Edgar Toschi-Dias, Maria Janieire N. N. Alves, Raffael F. Fraga, Jonathan C. Jun, Carlos E. Negrão, Eduardo M. Krieger, Vsevolod Y. Polotsky, Geraldo Lorenzi-Filho

**Affiliations:** 1 Heart Institute (InCor), University of São Paulo Medical School, São Paulo, Brazil; 2 Division of Pulmonary and Critical Care Medicine, Johns Hopkins University School of Medicine, Baltimore, Maryland, United States of America; Lerner Research Institute, United States of America

## Abstract

**Background:**

Obstructive Sleep Apnea (OSA) is tightly linked to some components of Metabolic Syndrome (MetS). However, most of the evidence evaluated individual components of the MetS or patients with a diagnosis of OSA that were referred for sleep studies due to sleep complaints. Therefore, it is not clear whether OSA exacerbates the metabolic abnormalities in a representative sample of patients with MetS.

**Methodology/Principal Findings:**

We studied 152 consecutive patients (age 48±9 years, body mass index 32.3±3.4 Kg/m^2^) newly diagnosed with MetS (Adult Treatment Panel III). All participants underwent standard polysomnography irrespective of sleep complaints, and laboratory measurements (glucose, lipid profile, uric acid and C-reactive protein). The prevalence of OSA (apnea-hypopnea index ≥15 events per hour of sleep) was 60.5%. Patients with OSA exhibited significantly higher levels of blood pressure, glucose, triglycerides, cholesterol, LDL, cholesterol/HDL ratio, triglycerides/HDL ratio, uric acid and C-reactive protein than patients without OSA. OSA was independently associated with 2 MetS criteria: triglycerides: OR: 3.26 (1.47–7.21) and glucose: OR: 2.31 (1.12–4.80). OSA was also independently associated with increased cholesterol/HDL ratio: OR: 2.38 (1.08–5.24), uric acid: OR: 4.19 (1.70–10.35) and C-reactive protein: OR: 6.10 (2.64–14.11). Indices of sleep apnea severity, apnea-hypopnea index and minimum oxygen saturation, were independently associated with increased levels of triglycerides, glucose as well as cholesterol/HDL ratio, uric acid and C-reactive protein. Excessive daytime sleepiness had no effect on the metabolic and inflammatory parameters.

**Conclusions/Significance:**

Unrecognized OSA is common in consecutive patients with MetS. OSA may contribute to metabolic dysregulation and systemic inflammation in patients with MetS, regardless of symptoms of daytime sleepiness.

## Introduction

Metabolic syndrome (MetS) constitutes a clustering of metabolic and cardiovascular abnormalities including central obesity, insulin resistance, dyslipidemia, and increased blood pressure in the same individual [1]. Despite some controversy, MetS is associated with higher cardiovascular risk than one might expect from simple addition of its individual components [2]–[4]. However, other factors may contribute to the high cardiovascular burden observed in patients with MetS.

Obstructive sleep apnea (OSA) is characterized by recurrent episodes of partial or complete obstruction of the upper airway, intermittent hypoxia and frequent arousals from sleep [5]. There is abundant evidence from humans and animals suggesting that OSA may impact every aspect of MetS, including obesity [6], hypertension [7], insulin resistance [8] and dyslipidemia [9], [10]. Furthermore, OSA and MetS have been previously shown to co-exist [11]–[20]. However, previous studies were limited by small sample size and/or selection bias, because they included patients referred for sleep studies due to sleep-related complaints. Thus, the prevalence and impact of OSA in consecutive patients with MetS have not been adequately explored. It is not certain whether the overlap between OSA and MetS is simply a result of underlying obesity, or if OSA represents an additional burden that exacerbates metabolic dysfunction and systemic inflammation in patients with MetS. The impact of daytime sleepiness on markers of cardiovascular risk in patients with MetS is unknown.

In the present investigation we enrolled consecutive patients with MetS with no previous diagnosis of OSA in order to evaluate if the presence of OSA is independently associated with (1) parameters of MetS, (2) parameters associated with cardiovascular risk but not included in the MetS definition. Moreover, we explored if effects of OSA on metabolic and inflammatory indices are modulated by the extent of daytime sleepiness.

## Materials and Methods

### Ethics Statement

The local Ethics Committee (Institutional Review Board – Heart Institute) approved the protocol, and all participants gave written informed consent.

### Patients

We studied consecutive patients with a recent diagnosis of MetS recruited from the Heart Institute (InCor) from October 2008 to December 2009. All participants were asymptomatic outpatients admitted for routine check-up evaluations. No sleep questionnaire was applied at the time of the recruitment. Patients with established cerebrovascular disease, coronary disease, heart failure, rheumatologic diseases, renal failure; hypothyroidism, pregnancy, history of smoking, and regular exercisers were excluded as well as patients with a previous diagnosis of OSA. In addition, we excluded patients who were using hypoglycemic medications, insulin, fibrates, statins, uricosuric agents (such as allopurinol), steroids and contraceptives. All participants underwent a detailed history and physical. The body mass index was calculated after body weight and height were measured in subjects wearing light clothing without shoes. Waist circumference was measured with soft tape on standing subjects midway between the lowest rib and the iliac crest. Two blood pressure recordings were obtained from the right arm of patients in a sitting position after 15 minutes of rest at 5-minute intervals, and their mean value was calculated. The diagnosis of hypertension was based on current guidelines [21].

### Blood samples

Fasting blood samples were drawn for determination of glucose, total cholesterol, low-density lipoprotein (LDL), high-density lipoprotein (HDL), triglycerides, and uric acid using enzymatic methods. High-sensitivity C-reactive protein was measured by using particle enhanced immunonephelometry (Dade Behring, Inc. Deerfield, Illinois). All samples were collected in the absence of clinical evidence of active infection/ inflammatory processes such as viral infections.

### Definition of metabolic syndrome

MetS was diagnosed according to the National Cholesterol Education Program, Adult Treatment Panel III (NCEP III) (1), if 3 of the 5 following factors were present: 1) waist circumference (≥102 cm in men and ≥88 in women), 2) triglycerides ≥150 mg/dL, or patient on specific drug treatment, 3) HDL <40 mg/dL in men and <50 mg/mg/dL in women, or when on specific drug treatment, 4) arterial blood pressure ≥130 or 85 mm Hg for systolic and diastolic blood pressure, respectively, or patient on antihypertensive drug treatment, and 5) fasting glucose ≥100 mg/dL or patient on specific drug treatment.

### Sleep Parameters

Within 1 month after blood sample collection, all participants underwent a standard overnight polysomnography as previously described [22]. Apnea was defined as complete cessation of airflow for at least 10 seconds, associated with oxygen desaturation of 3%. Hypopnea was defined as a reduction in respiratory signals for at least 10 seconds associated with oxygen desaturation of 3%. The apnea-hypopnea index (AHI) was calculated as the total number of respiratory events (apneas plus hypopneas) per hour of sleep. The AHI cutoffs for mild, moderate and severe OSA were 5 to 14.9, 15 to 29.9, and ≥30 events per hour of sleep, respectively. Because of a high expected prevalence of OSA in this population, the presence of OSA was also restricted to the moderate to severe cases, i.e., AHI ≥15 events per hour of sleep as previously described [18]. Daytime somnolence was evaluated by the Epworth sleepiness scale [23], with a score of >10 considered excessive daytime sleepiness.

### Statistical Analysis

Data were analyzed with SPSS statistical software version 18.0 (Chicago, Illinois, USA). The comparison of continuous variables between patients with and without OSA was performed using the Student *t* test or Mann-Whitney test, when appropriate. Categorical variables were expressed by frequency distribution and were compared using the Fisher exact test. Continuous variables with normal distribution were expressed as mean ±SD. Otherwise, they were presented as median (interquartile range). In order to analyze the relative role of OSA either as a categorical variable (presence or absence of OSA) or as a continuous one (AHI, minimum oxygen saturation during sleep and total sleep time below 90%) we performed two independent analyses: 1) Univariable and multivariable logistic regression analysis to model the association of each MetS criteria (waist circumference, triglycerides, HDL, blood pressure and glucose) and non-MetS risk factors (cholesterol/HDL ratio ≥4.5 [24], triglycerides/HDL ratio >3 [25], C-reactive protein >3 mg/L [26] and uric acid >7 mg/dL [27]) according to the presence of OSA (adjusted for age, sex, race, body mass index and waist circumference); 2) Multiple linear regression analysis (Stepwise linear regression analysis) to evaluate the relative role of markers of OSA severity with absolute values of components of MetS and metabolic and inflammatory variables not included in the MetS criteria (cholesterol/HDL ratio, uric acid and C-reactive protein). We used a P = 0.15 as the critical value for entering/excluding variables in the model. Independent variables were age, sex, race, body mass index, waist circumference and sleep parameters. In all multiple regression analysis we avoided the presence of multicollinearity. In a subgroup of patients with MetS and OSA, we also performed a comparison of the independent variables associated with the presence of OSA according to the presence or absence of excessive daytime sleepiness.

## Results

**Figure 1:**
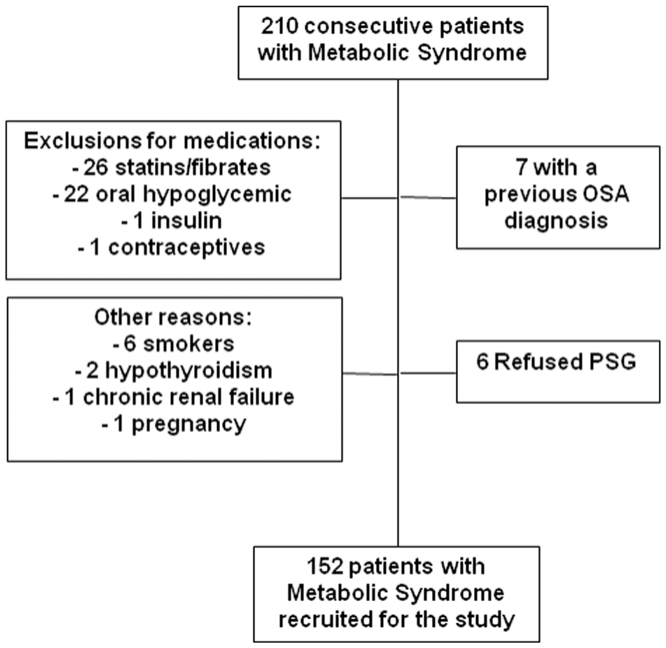
Patient flow diagram. Some patients had multiple exclusions reasons. OSA: Obstructive Sleep Apnea. PSG: Polisomnography.

We initially enrolled 210 consecutive patients with a recently confirmed diagnosis of MetS. The final sample was comprised of 152 patients, because 58 subjects met one or more exclusion criteria (Figure 1). Characteristics of patients including age, sex, body mass index and waist circumference were not different between patients included and excluded from the study (P>0.2 for all comparisons). Forty participants (26.3%) were involved in the previous study evaluating the impact of OSA on markers of atherosclerosis in consecutive patients with MetS [28]. Notably only 7 (3.3%) were excluded from the initial screening because of a previous diagnosis of OSA. The prevalence of OSA (AHI ≥15 events per hour of sleep) in patients with MetS was 60.5%.

**Figure 2:**
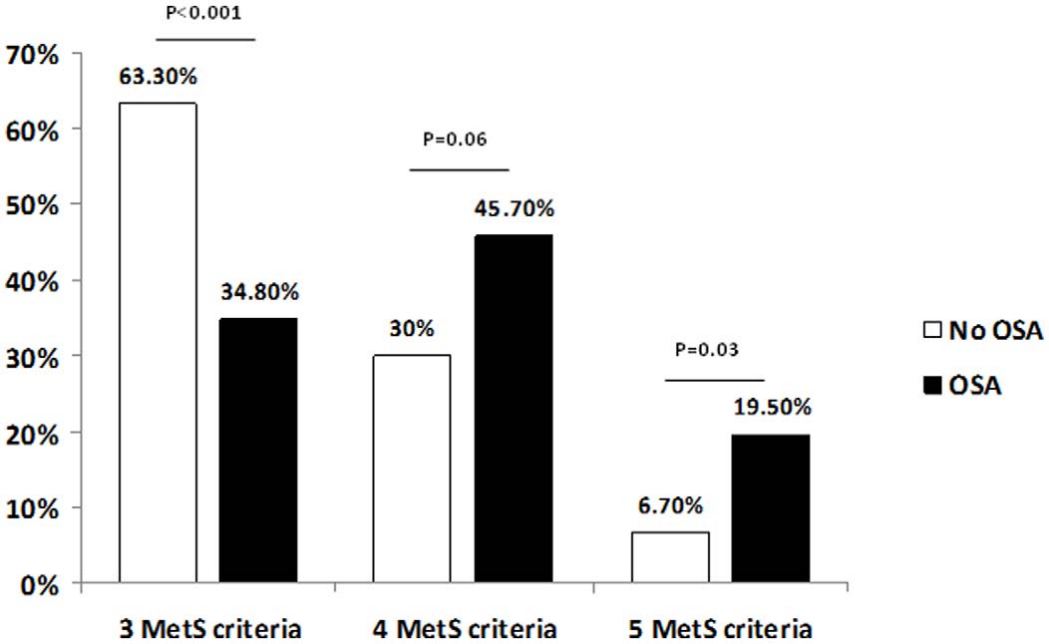
Rate of MetS criteria in patients with and without OSA.

**Figure 3:**
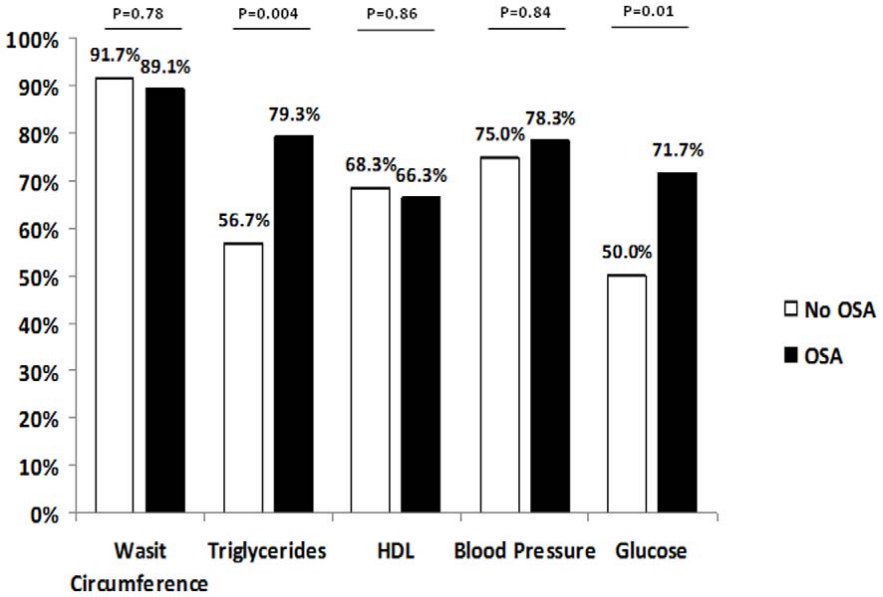
Frequency of each MetS criteria between groups (B).

Twenty seven patients (17.8%) presented with an AHI <5 events per hour of sleep. Thirty three patients (21.7%) presented with an AHI from 5 to 14.9 events per hour of sleep. Moderate OSA (AHI 15–29.9 events per hour of sleep) and severe OSA (AHI ≥30 events per hour of sleep) were observed in 34 (22.4%) and 58 patients (38.1%), respectively. The demographic, anthropometric and sleep characteristics of the total population of patients studied, as well as comparisons of patients with or without OSA are shown in Table 1. Overall, the present sample included middle age patients. As expected, the great majority of participants were obese with high waist circumference measurements. There were no significant differences in sex, race, body mass index, waist circumference, hypertension, and diabetes status between patients with and without OSA. The anti-hypertensive medications were also similar between hypertensive patients with and without OSA: diuretics (75% vs. 72%), beta-blockers (38% vs.55%), calcium channel blockers (44% vs. 34%), angiotensin-converting enzyme inhibitors (64% vs. 50%) and angiotensin II receptor blockers (22% vs. 17%), were similar (P>0.1 for all comparisons). MetS patients with OSA were older (Table 1). Patients with OSA met a higher number of MetS criteria due to the higher rate of hypertriglyceridemia and hyperglycemia than patients without OSA (Figure 2 and 3). Patients with OSA and MetS had significantly higher levels of MetS-defining parameters including blood pressure, fasting blood glucose and serum triglycerides compared to patients with MetS alone (Table 2). The levels of HDL were similar between patients with and without OSA. In contrast, the levels of several non-MetS parameters including serum total cholesterol, LDL, triglycerides/HDL ratio, cholesterol/HDL ratio, uric acid and C-reactive protein were also higher in patients with OSA.

**Table 1:** Patient characteristics.

	Total Sample (n = 152)	No OSA (n = 60)	OSA (n = 92)	*P value* *
**Age (years)**	48±9	46±8	49±8	0.02
**Males (%)**	62.5	55	67	0.13
**Caucasians (%)**	74	70	77	0.34
**Body mass index (kg/m^2^)**	32.3±3.4	32.2±3.8	32.3±3.2	0.76
**Hypertension (%)**	66.4	61.7	69.6	0.38
**Diabetes (%)**	7.9	9.1	8.2	1.00
**Number of MetS criteria (n)**	3 (3−4)	3 (3−4)	4 (3−4)	<0.01
**Apnea-hypopnea index (events/hour)**	19.6 (7.1−39.0)	5.0 (2.6−8.8)	34.7 (25.0−54.3)	<0.001
**Lowest oxygen saturation (%)**	83 (74−87)	87 (85−90)	77 (69−84)	<0.001
**Total sleep time oxygen saturation <90% (%)**	3.0 (0.2−10.0)	0.3 (0−1.8)	8.9 (2.7−24.5)	<0.001
**Epworth sleepiness scale**	10 (8−11)	9 (8−10)	10 (8−12)	0.03
**Excessive daytime sleepiness,† n (%)**	47 (30.9)	14 (23.3)	33 (35.9)	0.11

*For comparisons between MetS patients with and without OSA. †Epworth sleepiness scale score >10.

MetS: Metabolic Syndrome. OSA: Obstructive Sleep Apnea. Variables with normal distribution are expressed as mean±SD. Variables with skewed distribution are presented as median (interquartile range).

**Table 2:** Quantitative values of metabolic and inflammatory profile in Metabolic Syndrome patients with and without Obstructive Sleep Apnea.

	Total Sample (n = 152)	No-OSA (n = 60)	OSA (n = 92)	*P value* *
***Variables included in MetS criteria***				
** Waist circumference (cm)**	105.1±8.2	104±9	106±8	0.16
** Triglycerides (mg/dL)**	196±86	170±81	213±85	0.002
** HDL cholesterol (mg/Dl)**	39 (34−47)	39 (33−47)	38 (34−47)	0.83
** Systolic blood pressure (mm Hg)**	139±24	134±22	142±24	0.03
** Diastolic blood pressure (mm Hg)**	84±14	81±14	86±15	0.05
** Fasting glucose (mg/dL)**	102±11	98±10	105±11	<0.001
***Variables not included in MetS criteria***				
** Total cholesterol (mg/dL)**	216±39	204±32	223±36	0.003
** Total cholesterol/HDL ratio**	5.5±1.4	5.1±1.4	5.7±1.5	0.02
** Triglycerides/HDL ratio**	5.1±2.9	4.4±2.5	5.6±2.9	0.009
** Uric acid (mg/dL)**	6.5±1.4	5.8±1.3	6.9±1.4	<0.001
** C-reactive protein (mg/dL)**	3.0 (2.0−4.0)	2.6 (1.3−3.2)	3.9 (2.7−4.4)	<0.001

*For comparisons between patients with and without OSA.

Univariable and multivariable logistic regression analysis (Table 3) showed that the presence of OSA was independently associated with 2 of 5 criteria for MetS (triglycerides and glucose). Moreover, the presence of OSA was independently associated with abnormally elevated cholesterol/HDL ratio, uric acid and C-reactive protein. There was a strong trend for an independent association between the presence of OSA and triglycerides/HDL ratio.

**Table 3:** Univariable and multivariable logistic regression analysis for the association between presence of OSA with variables included and not included in the MetS criteria.

	Unadjusted odds ratio (95% CI)	Adjusted odds ratio (95% CI)*	Adjusted P value
***Variables included in MetS criteria***			
** Waist circumference criteria**	0.75 (0.24−2.30)	0.73 (0.21−2.50)	0.61
** Triglycerides criteria**	2.94 (1.43−6.02)	3.26 (1.47−7.21)	0.004
** HDL-C criteria**	0.91 (0.46−1.83)	0.87 (0.42−1.80)	0.71
** Arterial blood pressure criteria**	1.20 (0.56−2.58)	1.02 (0.45−2.32)	0.96
** Fasting glucose criteria**	2.54 (1.29−5.01)	2.31 (1.12−4.80)	0.02
***Variables not included in MetS criteria***			
** Total cholesterol/HDL ratio ≥4.5**	2.40 (1.17−4.91)	2.38 (1.08−5.24)	0.03
** Triglycerides/HDL ratio >3**	2.21 (1.04−4.68)	2.19 (0.95−5.04)	0.07
** Uric Acid >7 mg/Dl**	4.18 (1.78−9.82)	4.19 (1.70−10.35)	0.002
** C-reactive protein >3 mg/L**	4.92 (2.41−10.03)	6.10 (2.64−14.11)	<0.001

*Adjusted for age, sex, race, body mass index and waist circumference (except for waist circumference criteria).

Multiple linear regression analysis showed that the AHI or minimum oxygen saturation during sleep were independently associated with serum levels of triglycerides and glucose as well as with several metabolic and inflammatory parameters not included in the MetS criteria (cholesterol/HDL ratio, uric acid and C-reactive protein - Table 4). Although C-reactive protein has a skewed distribution, the residuals from this model were normally distributed.

**Table 4:** Stepwise linear regression analysis for the association between markers of OSA severity and components of MetS and metabolic/inflammatory variables not included in the MetS criteria*.

	Variables	Coefficient (β)	95% CI	P value
***Variables included in MetS criteria***				
** Triglycerides**	Race	−14.530	−27.930 −1.150	0.03
	Body mass index	−5.670	−10.740 −0.600	0.03
	Minimum O_2_ saturation	−2.079	−4.120−0.040	0.04
** Glucose**	Age	0.315	0.108 0.522	0.003
	Apnea-hypopnea index	0.081	0.014 0.148	0.02
***Variables not included in MetS criteria***				
** Cholesterol/HDL ratio**	Apnea-hypopnea index	0.013	0.004 0.022	0.006
** Uric Acid**	Sex	0.845	0.349 1.342	0.001
	Apnea-hypopnea index	0.013	0.002 0.024	0.02
** C-reactive protein**	Minimum O_2_ saturation	−0.042	−0.072−0.013	0.005
	Sex	−0.632	−1.249−0.002	0.04
	Age	0.036	0.003 0.070	0.03

*Variables used in the model: age, sex, race, body mass index, waist circumference and sleep parameters (apnea-hypopnea index, minimum O_2_ saturation during sleep and total sleep time below 90%).

Systolic and diastolic blood pressure were independently related only to age (data not shown; P<0.001 for both comparisons).

**Figure 4:**
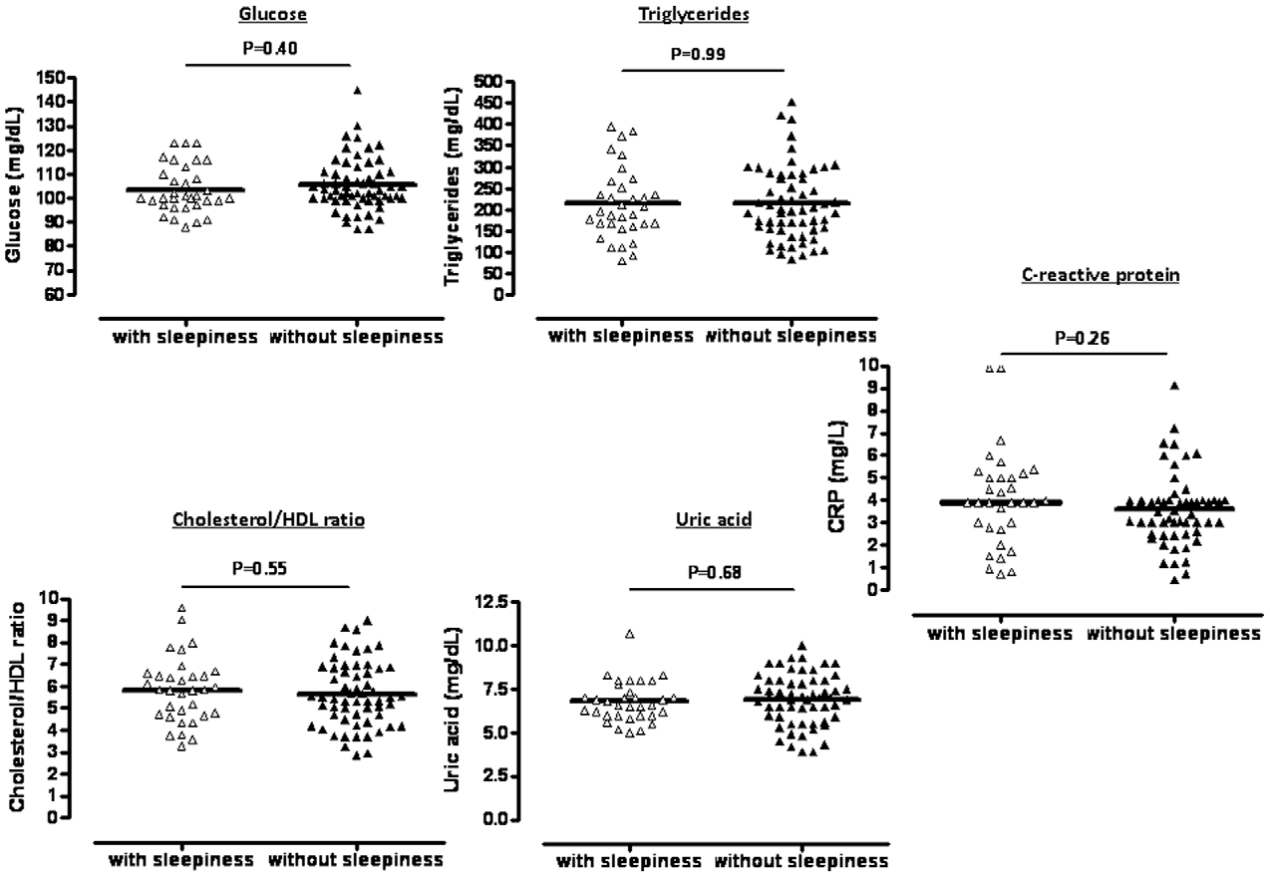
Levels of glucose, triglycerides, cholesterol/HDL ratio, uric acid and C-reactive protein in patients with MetS and OSA according to the presence or absence of excessive daytime sleepiness.

Figure 4 shows that the independent variables associated with OSA (glucose, triglycerides, cholesterol/HDL ratio, uric acid and C-reactive protein) were similar in patients with and without excessive daytime sleepiness.

## Discussion

The present study showed that, in consecutive patients with MetS, unrecognized OSA is common and independently associated with biomarkers of metabolic dysfunction and systemic inflammation. Specifically, OSA was associated with two MetS criteria, triglycerides and glucose; and with three non-MetS cardiovascular risk factors, cholesterol/HDL ratio, uric acid, and C-reactive protein. These associations were not influenced by the presence or absence of excessive daytime sleepiness. Taken together, our data suggest that OSA may contribute to the metabolic and cardiovascular burden of patients with MetS.

Previous clinical studies of MetS failed to consider OSA as a potential confounding factor that contributes the cardiovascular risk [29], [30]. Accordingly, the American Heart Association Scientific statement on MetS briefly discussed OSA and classified it as being of interest to “other fields of medicine,” giving it the same attention given to cholesterol gallstones and lypodystrophies [1]. Conversely, the majority of previous investigations derived from the sleep community were intrinsically biased studying only patients referred for sleep studies [11]–[16]. Our study design allowed us to systematically examine the prevalence of unrecognized OSA in consecutive sample of patients with MetS. We found a 60.5% prevalence of OSA in MetS, even using conservative diagnostic OSA criteria (AHI ≥15 events per hour of sleep). The prevalence of OSA observed in this study is in line with two previous reports from different groups (ranging from 68 to 87.5%) that evaluated patients with MetS [17], [18]. Consistently with our data, a recent report found a high prevalence of OSA (∼86%) in obese patients with type 2 diabetes [31]. The high prevalence of OSA among our consecutive patients with MetS may be due to the shared feature of visceral obesity in both syndromes. In fact, visceral rather than the subcutaneous or total body fat predisposes to the development of OSA [32]. However, the effect of OSA on metabolic function in MetS, at least in terms of glucose control, has been shown to occur independently of waist circumference, a surrogate marker of visceral adiposity [18]. In the present larger study, which excluded patients treated with hypoglycemic and lipid-lowering medications, we show that the co-existence of OSA in patients with MetS is associated with increased glucose and triglycerides levels.

We have also shown that OSA is associated with non-MetS cardiovascular risk markers, including cholesterol/HDL ratio, uric acid, and C-reactive protein. These biomarkers have validated as indices of the cardiovascular risk. A large prospective study showed that a cholesterol/HDL ratio >4.5 is a better predictor of ischemic heart disease than total cholesterol, HDL cholesterol, or non-HDL cholesterol [33]. Uric acid, the catabolic end product of ATP, is associated with oxidative stress, inflammation, subclinical atherosclerosis, and an increased risk of cardiovascular events [34]–[36]. Independent studies have shown that plasma uric acid is often elevated in subjects with the MetS and OSA [37], [38]. Our study suggests that OSA has an additive effect on uric acid levels in patients with MetS. Finally, pro-inflammatory effects of MetS and OSA were widely discussed in the literature and many independent studies reported that both MetS [26] and OSA [39] are independently associated with high C-reactive protein levels, which is a marker of cardiovascular inflammation. C-reactive protein adds clinically important prognostic information to the MetS [26]. Collectively, our study suggests that the severity of OSA is independently associated with unfavorable lipid profile, hyperuricemia and inflammation in consecutive patients with MetS.

The independent association of OSA with dyslipidemia and systemic inflammation observed in our study has biological basis. Intermittent hypoxia, the hallmark of OSA, causes dyslipidemia in mice by up-regulating hepatic lipid biosynthesis and lipoprotein secretion via hypoxia inducible factor 1 alpha [9], [10]. Intermittent hypoxia also activates pro-inflammatory transcription factors such as nuclear factor kappa B that promote activation of various inflammatory cells with the downstream consequence of expression of pro-inflammatory mediators that may lead to endothelial dysfunction [40].

In the present study, we found that several metabolic and inflammatory markers associated with OSA were similar in patients with and without excessive daytime sleepiness. Other investigators have also shown that OSA is associated with markers of atherosclerosis [28] and mortality irrespective of daytime symptoms [41]. These collective results challenge the notion that only sleepy patients with OSA are at increased cardiovascular risk. OSA may not be suspected in non-sleepy persons, and, therefore, overlooked as a potential cardiovascular risk factor.

The main strength of our study is the study design, since consecutive subjects with MetS underwent full polysomnography, regardless of their sleep complaints. The main limitations are: First, the present study comprised a relatively small number of participants. The observation of a nonsignificant trend of an independent association between OSA and triglicerydes/HDL ratio, a marker of insulin resistance, may be due to the lack of power to detect this difference. Therefore, the impact of OSA in patients with MetS may be even greater. Second, our patients were middle-aged and without a history of coronary disease and stroke or intake of statins, fibrates and hypoglycemic drugs. Hence, our results may not be applicable to other age groups, or patients with established cardiovascular disease. On the contrary, the exclusion of drugs that directly affect the metabolic and inflammatory profile in MetS may be an advantage for studying metabolic and pro-inflammatory effects of OSA. Third, we were unable to exclude patients on anti-hypertensive treatment, since more than 50% of patients were on medications, which could not be discontinued for ethical reasons. Finally, the cross sectional nature of the study does not prove cause-effect relationships between OSA and metabolic and inflammatory markers.

In conclusion, we have shown that OSA is highly common in patients with MetS. OSA is independently associated with increased prevalence and severity of hypertriglyceridemia and hyperglycemia, as well as with several other markers of metabolic and inflammatory dysregulation (cholesterol/HDL ratio, uric acid and C-reactive protein). Our data strongly suggest that patients with MetS need to be evaluated for OSA regardless of daytime sleepiness. We hypothesize that the presence of OSA can exacerbate MetS and further increase cardiovascular morbidity and mortality. Future interventional studies will demonstrate whether treatment of OSA will improve MetS and cardiovascular outcomes in these patients.
